# Study on Local High-Velocity-Impact Characteristics of Carbon Fiber Composite Laminates Based on Experimental Image Sequences

**DOI:** 10.3390/ma18081833

**Published:** 2025-04-16

**Authors:** Shuguang Yao, Minxin Zhou, Jie Xing

**Affiliations:** 1 Key Laboratory of Traffic Safety on Track School of Traffic & Transportation Engineering, Central South University, Ministry of Education, Changsha 410075, China; ysgxzx@csu.edu.cn (S.Y.); 224211058@csu.edu.cn (M.Z.); 2 Joint International Research Laboratory of Key Technology for Rail Traffic Safety, Central South University, Changsha 410075, China; 3 National & Local Joint Engineering Research Center of Safety Technology for Rail Vehicle, Central South University, Changsha 410075, China

**Keywords:** carbon fiber composite laminates, high-velocity impact, impact response

## Abstract

For the complex operating environment of rail vehicles impacted by foreign objects, this study carried out experiments on carbon fiber composite laminates impacted by projectiles of three different materials in the gas gun apparatus. High-speed photography was used to capture the impact dynamic process, with subsequent analysis of image sequences enabling the quantification of both projectile energy characteristics and laminate mechanical responses. The key findings reveal that (1) the impact force demonstrates a nonlinear relationship with the energy input, exhibiting diminishing growth rates at higher energy levels, while deformable aluminum projectiles yield significantly lower average impact forces; (2) crack propagation follows a characteristic pattern of initial expansion with the impact energy reaching maxima of 88.40 mm (concrete), 41.48 mm (steel), and 80.35 mm (aluminum), and then decreasing, followed by stabilization post-perforation; (3) the penetration depth showed an accelerating nonlinear growth trend with progressively higher rates of increases as the impact energy rose, and its variation trend exhibited a correlation with the crack length. The results provide quantitative insights into carbon fiber laminates’ damage mechanisms, which offer practical implications for the composite structure design in transportation systems subjected to foreign object impacts.

## 1. Introduction

Carbon fiber composites have high specific strength, a high specific modulus, fatigue resistance, and corrosion resistance [1,2,3], making them popular materials for manufacturing high-strength, rigid structures and equipment, and they are widely used in lightweight engineering applications [4,5,6,7]. In the field of rail transit, carbon fiber composite laminates have been tried for use in manufacturing internal facilities and car body structures such as drivers’ cabs, bogies, roofs, and the equipment compartments of rail vehicles [8,9], which has greatly reduced the weight of the car body. However, in the process of high-velocity operation, rail vehicles face complex external environments, such as snow and hail encountered in operation, rockfall and ice at tunnel mouths, and ballast [10] and other foreign bodies with an influence on the high-velocity impact of train vehicles. When carbon fiber composites are applied to rail vehicles to achieve lightweight engineering applications, their safety after being impacted needs to be further demonstrated. Unlike traditional metal materials, the failure mechanism of composite laminates after impact is highly complex and difficult to predict in terms of damage development. For structures with local and non-severe damage, repair and reuse can achieve the purpose of reducing maintenance costs and extending service life [11]. Existing studies show that repaired carbon fiber composite plates still maintain relatively good post-impact performance [12,13]. However, the repair threshold of damage defects is difficult to determine due to complex failure modes. Therefore, it is necessary to carry out research on the damage response of carbon fiber composite laminates under impact conditions.

Many scholars have studied the behavioral response of composite laminates under low-velocity-impact conditions. Low-velocity impact is usually carried out using a drop-weight-testing machine, and the response results such as impact load and absorbed energy can be obtained through the displacement–time curve of the punch, and the damage results can be detected by non-destructive testing methods such as an optical microscope [14,15], ultrasonic detection [16], and X-ray scanning [17]. Falcó et al. [18] analyzed that the kinetic energy of an impactor under low-velocity impact is mainly dissipated through different failure mechanisms such as friction between the punch and laminate, fiber fractures, the impact surface indentation, the interlayer delamination, and the matrix tensile cracking caused by local stress. Pai et al. [19] analyzed the force–time curve and concluded that the sample delamination and fiber fractures caused by the rapid expansion of matrix cracks in the thickness direction reduced the stiffness of the laminates, and thus the maximum load decreased rapidly. Damghani et al. [20] agreed that the jagged fluctuation in the loading phase was due to the generation of microcracks in the matrix, but there was an obvious bimodal load during the impact process, and it was explained that this was because of the delamination failure of the specimen when there was still residual kinetic energy in the punch. Liu et al. [21] believed that the duration of contact between the impactor and the sample was independent of the impact energy in the low-velocity-impact test, and the braid structural laminate could limit the damage propagation in a smaller area compared with the unidirectional laminate.

However, for carbon fiber laminates used in rail vehicles, in addition to the low-velocity impact of foreign bodies during operation, they will also encounter more dangerous high-velocity impact. Kazemi et al. [22] divided the fiber-reinforced polymer laminate penetrated by high-velocity impact into three regions along the thickness direction, namely the crushing and fragmentation zone on the front side of the impact, the shear plugging zone at the center of the layer and the distal end tensile failure, and the delamination zone. High-velocity-impact experiments are usually performed using gas gun equipment. Cai et al. [23] studied the high-velocity-impact resistance of the CFRP and GLARE laminated structures, and the results showed that the main damage of the two laminates was fiber damage and plastic deformation, respectively. Zhu et al. [24] studied the residual compressive capacity of the CFRP laminates after high-velocity impact and found that the residual strength dropped to the minimum near the ballistic limit, and the failure modes at this time were mainly stratification and buckling. At present, most of the studies on the high-velocity impact of composite laminates focus on the energy absorption rate and ballistic limit of the panels, and the analysis of the damage response of high-velocity impact but the non-penetration stage is insufficient. Moreover, due to the failure mode of carbon fiber laminates and the experimental equipment of projectile impacts, it is very difficult to obtain the mechanical response in high-velocity-impact experiments.

There are many factors that affect the impact response of composite laminates. There are internal factors such as the composite fiber type and laminate structure, and external factors such as the impact energy and projectile type will affect the impact resistance of the laminate. A number of studies have been carried out on the effect of the structure of composite laminates on impact properties. Claus et al. [25] investigated the impact response of composites with different fiber types and matrices in the case of penetration, and the experimental results showed that the composite with epoxy resin as the matrix showed cross-shaped cracks after penetration, while the polypropylene composites showed an X-shape and absorbed more energy. According to the study of Zhao et al. [26], a unidirectional fiber laminate had a higher critical penetration velocity, but it mainly absorbed energy through interlayer delamination damage, which could lead to larger deformation, while woven and braided laminates showed higher integrity after impact by absorbing energy through multiple failure modes. Seamone et al. [27,28] analyzed carbon fiber composite laminates with different thicknesses. The results indicated that the delamination area was positively correlated with the thickness of the laminate. The crater depth of thin plates increased linearly with impact energy, while that of thick plates was related to the occurrence of the delamination damage behavior of the laminate.

The influence of different impact conditions and ejector forms on the impact response of composite laminates is also being explored. Pernas-Sánchez et al. [29] proposed a simplified energy loss model for positive and oblique impacts and found that there were differences in the energy absorption mechanisms at different impact angles, and the crushing damage of oblique impact is greater than that of the vertical positive impact carbon fiber laminates. Carrasco et al. [30] investigated the impact angle using numerical analysis and also examined the individual and joint effects of laminate clamping stress, the laminate thickness to projectile diameter ratio, and the laminate density to projectile density ratio on residual stresses and ballistic limits. Zhang et al. [31,32] studied different impact positions and impact angles, and it was believed that the laminate would produce asymmetrical deformation under the condition of edge impact, but when the impact velocity was increasing the local effect of the impact force in a more obvious way, the difference between the center impact and the edge impact became smaller. Vijayan et al. [33] used experiments and numerical simulations to study the effect of the top angle of a conical projectile on the impact deformation, local failure mode, and ballistic limit. And the 45° top angle had the strongest ability to penetrate the target plate, and the impact energy required for penetration was minimal. Liu et al. [34] investigated the effect of cylindrical projectiles with a different hardness on the impact response of CF/PEEK laminates, and the impact of high-density polyethylene projectiles had greater impact deflection than that of gelatin projectile laminates and left more pronounced impact damage on the laminate surface. The impact experiments conducted by Sangsefidi et al. [35] showed that the degree of deformability of the projectile was related to the ballistic limit and the absorbed energy of the shot hole, with the rigid projectile possessing the smallest penetration velocity and the deformable projectile causing larger fiber breakage and permanent deformation of the laminate. In the existing studies on the influence of projectile materials, most metal projectiles were usually experimented with, but there were not only metal foreign bodies in the operating environment of rail vehicles but also other kinds of foreign body impacts.

While existing research has explored high-velocity impacts on CFRP composites, few studies have addressed the correlation between the impact energy and damage behavior modes of composite materials under conditions relevant to rail vehicles. Furthermore, the influence of projectile material properties—particularly comparisons between metallic and non-metallic projectiles—on damage responses remains poorly analyzed. This knowledge gap impedes the effective design and optimization of CFRP components for engineering applications requiring impact-resistant yet lightweight structural solutions. Our study aims to elucidate how the impact energy and projectile material affect damage responses in CFRP laminates under high-velocity impact.

This study employs a gas gun apparatus to conduct high-velocity-impact experiments on carbon fiber composite laminates, systematically examining damage responses under dynamic loading using projectiles of three different materials (concrete, steel, and aluminum). The effects of the impact energy and projectile material characteristics on structural responses are rigorously analyzed, with a focus on critical mechanical and damage responses including impact force, energy absorption, failure modes, crack length, penetration depths, and the correlation between responses. The experimental results can provide a reference for the design of the impact damage tolerance of carbon fiber composite laminates applied to cladding structures in the rail transit industry.

## 2. Experiments

### 2.1. Experimental Specimens

#### 2.1.1. Carbon Fiber Composite Laminates

**Figure 1:**
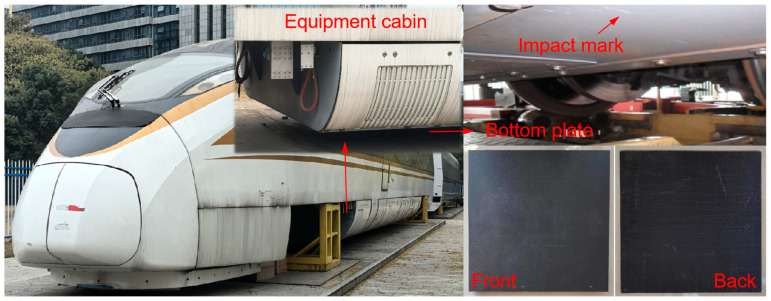
Carbon fiber specimen’s impact front and back.

The experimental specimens were made of 3 K and 12 K carbon-fiber-woven cloth and flame-retardant epoxy resin as the substrate, as shown in Figure 1, and they were formed by a vacuum infusion process, which could effectively reduce the porosity defects between fiber layers. The geometric size of the carbon fiber laminate was 400 × 400 × 5.2 mm, and the ply angle of the carbon fiber was [45/0] ns. The thickness of each layer was 0.4 mm, and detail information is shown in Table 1, and the density of the laminate was 1.55 g/cm^3^.

#### 2.1.2. Projectiles

In order to study the impact resistance of carbon fiber composite laminates against different materials and different volumes of impacts, three solid-sphere projectiles made of concrete, stainless steel, and aluminum were selected to carry out high-velocity-impact experiments on carbon fiber specimens, and the mass and size parameters of each projectile are shown in Table 2.

### 2.2. Experimental Setup

**Figure 2:**
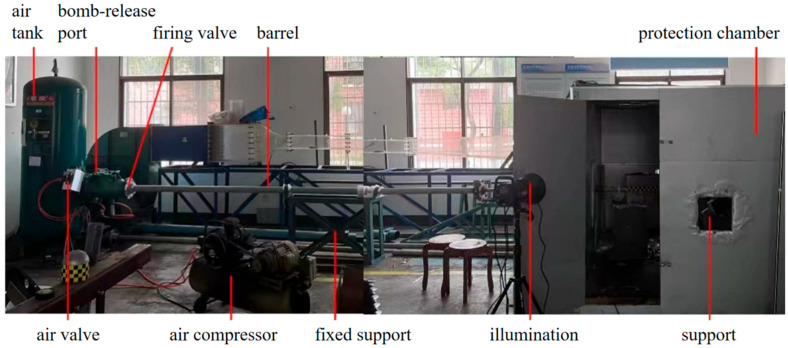
Diagram of experimental device.

The complete set of experimental equipment includes several parts, such as the gas gun power launch device, the gun barrel device, the fixed protection device, and the high-speed photography device, as shown in Figure 2. The gas gun power launcher uses an air compressor to regulate the air pressure in the air tank and realizes the different impact energies of the projectile by setting the pressure difference. For projectiles of different diameters, a precise barrel with a corresponding inner diameter should be selected to reduce the leakage of the air. The gas gun system incorporated a pressurized air tank capable of maintaining 0–1.37 MPa, accompanied by a modular barrel assembly which consisted of a fixed 2 m primary barrel and a changeable 1 m secondary barrel designed to accommodate projectiles of varying geometries. In the present study, inner diameters of 19 mm and 27 mm were employed for steel and aluminum/concrete projectiles, respectively, through barrel customization.

**Figure 3:**
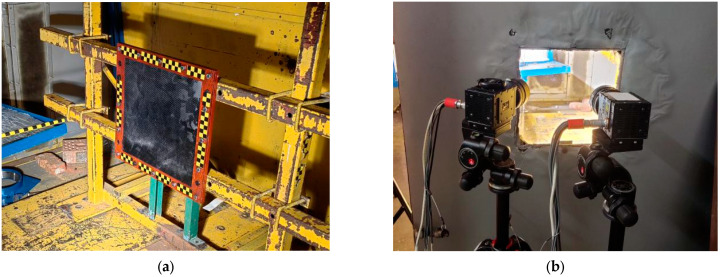
Diagram of (**a**) fixed protection device and (**b**) high-speed photography device.

As shown in Figure 3a, the carbon fiber laminate specimen was secured using a steel frame with 16 grade 8.8 M10 bolts, 12 bolts uniformly distributed along the four edges (3 bolts per edge, spaced at 100 mm intervals), and 4 additional bolts at each corner to prevent edge lifting. Each bolt was tightened to a controlled torque of 50 N·m using a calibrated torque wrench, and the equivalent clamping force was 25 kN. The theoretical maximum equivalent stress of the bolt was 318 MPa.

High-speed photographic recording was used to calculate the velocity of the projectile and to capture the full dynamics of the laminate impact, and the arrangement is shown in Figure 3b.

### 2.3. Experimental Results

Nine high-velocity-impact experiments with different velocities were performed for each projectile type, with a total of 27 sets of experimental results. The impact process is shown in Table 3, and the impact velocity distribution of three projectiles is shown in Table 4. The acquisition of projectile impact velocity will be described in Section 3.

## 3. Impact Characteristics Analysis Based on Impact Image Sequences

In this paper, the experimental results were processed by obtaining the impact velocity of the projectile, the rebound velocity, the accelerated speed, and the average impact force applied to the carbon fiber laminate from image sequences of high-speed photography.

### 3.1. Velocity Acquisition

**Figure 4:**
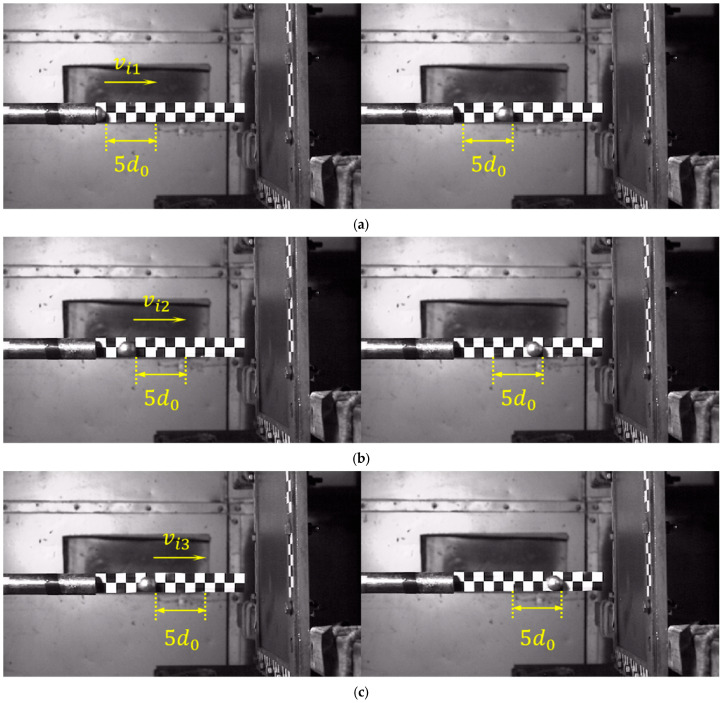
Diagram of impact velocity (**a**) first (**b**) second (**c**) third measurement process.

The high-speed photographic device, operating at 20,000 frames per second, recorded the entire process of the projectile impacting the carbon plate at high-velocity, and subsequently rebounding. The calibration paper positioned at the barrel exit enabled the measurement of both the impact velocity and residual rebound velocity. The projectile’s traversal across five calibration grids was measured, as shown in Figure 4a, and the corresponding frame count difference in the high-speed footage was determined to be $f_{i1}$; the velocity during this phase can be computed using the formula as follows:

(1) $$v_{i1}=\frac{5d_{0}}{f_{i1}F_{r}}$$

where $d_{0}$ = 15 mm represents the length of a single calibration marker, and $F_{r}$ = 1/20,000 s denotes the frame interval (reciprocal of the frame rate) of the high-speed camera system.

To minimize experimental errors, each set of measurements was conducted three times at different positions, yielding velocity $v_{i2}$ and $v_{i3}$, as illustrated in Figure 4b,c. The incident velocity of the projectile upon impacting the target plate was determined by calculating the average of the three measured velocities, expressed by the formula as follows:

(2) $$v_{i}=\frac{v_{i1}+v_{i2}+v_{i3}}{3}$$

Similarly, the rebound velocity of the projectile was determined by conducting three measurements at different points and calculating their average. Using this methodology, the impact and rebound velocities for 27 sets of experimental trials were obtained, with the results summarized in Table 5.

### 3.2. Impact Force Acquisition

**Figure 5:**
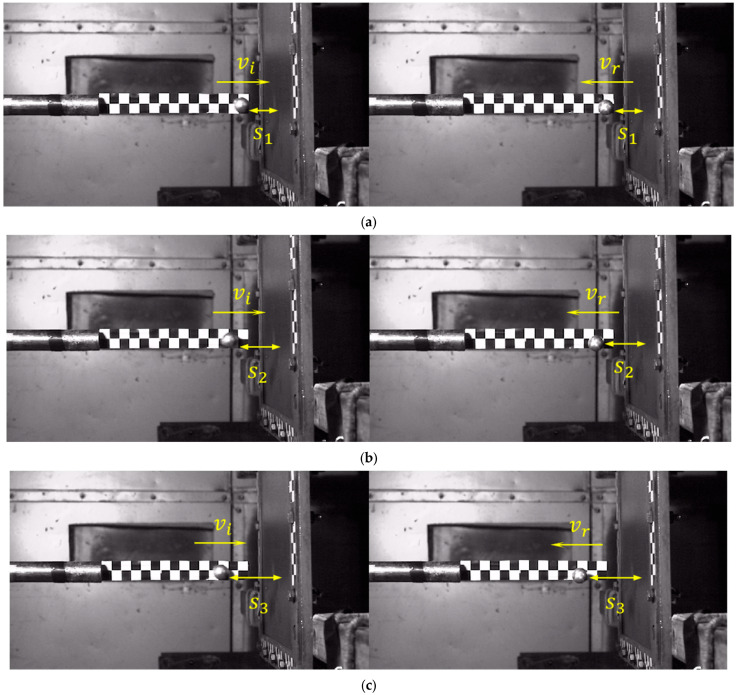
The process of the 20 g aluminum ball’s impact through (**a**) *s*_1_, (**b**) *s*_2_, (**c**) *s*_3_.

In past research of the high-velocity-impact composite laminate of gas guns [36,37], the impact force of the plate at the impact point was hard to obtain by a force-measuring instrument. Therefore, this paper determined the average impact force on the laminate based on Newton’s second law by calculating the acceleration. As illustrated in Figure 5a, the projectile traveled through distance *s*_1_ with an impact velocity *v_i_* and a rebound velocity *v_r_*, taking times *t_i_* and *t_r_*, respectively. This process was captured in high-speed photography with a frame difference in *f*_Δ_. The acceleration *a* of the projectile upon impact with the laminate is given by the following:

(3) $$a=\frac{∆v}{∆t}=\frac{\vec{v_{i}}-\vec{v_{r}}}{f_{∆}F_{r}-t_{i}-t_{r}}$$

To minimize errors, two additional segments of the trajectory were selected, as shown in Figure 5b,c. The average value of three calculations was used to determine the projectile acceleration, and the impact force *F* exerted on the laminate was calculated, according to Newton’s second law, as *F* = *ma*. Consequently, the average impact forces from 27 experimental sets were obtained and are summarized in Table 5.

**Figure 6:**
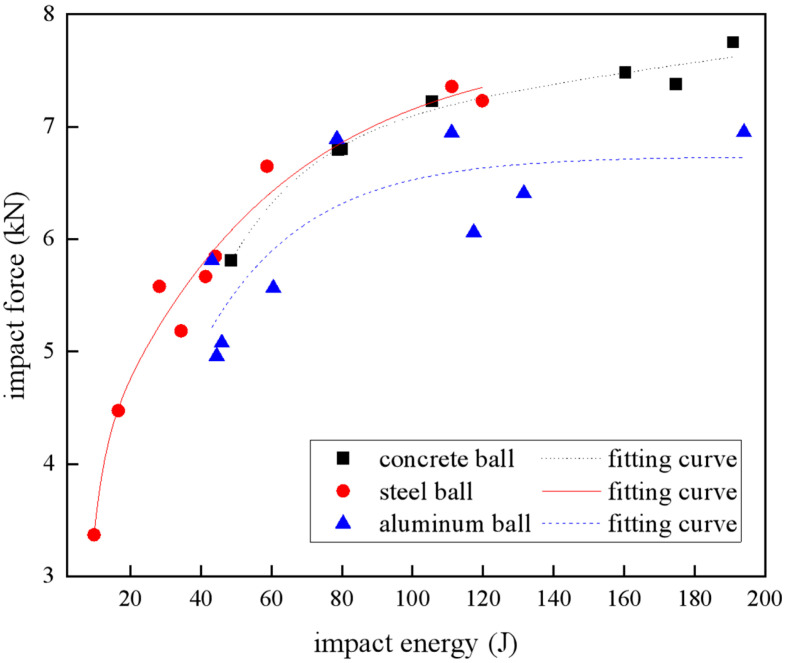
Impact force under different impact energies.

**Figure 7:**
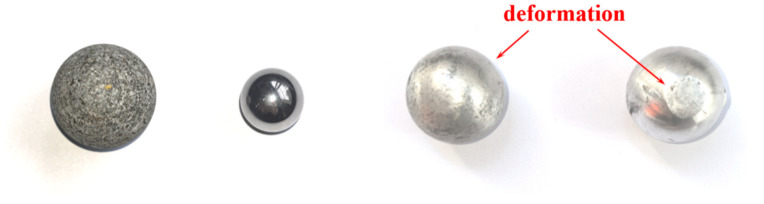
Projectile post-impact morphology.

Figure 6 shows the impact force of the laminate under different impact energies. There was a nonlinear relationship between the impact energy and the impact force, and the impact force on the specimen increased with the increase in impact energy, but the increasing trend gradually flattened. Thus, using Origin 2024’s Exponential Association (ExpAssoc) model to fit, the fitting parameters were recorded in Table 6. The regression analysis demonstrated excellent goodness of fit for the impact force–energy relationship in both the concrete and steel projectiles, with R^2^ exceeding 0.97. Notably, though aluminum projectiles exhibited a comparatively weaker correlation, the overall trend followed the fitting curve. Wherein, compared with the other two material projectiles, the laminate was subjected to a smaller impact force under the impact condition of the aluminum ball, which was due to the fact that the aluminum ball was more likely to be deformed during the collision, as shown in Figure 7, and the impact of the projectile after deformation changed from the point contact to the surface contact, and this made its contact time with the laminate longer, resulting in a smaller impact force. The impact energy of the undeformed concrete balls and steel balls was hardly converted into internal deformation energy, and the energy was directly transferred to the carbon fiber laminate, resulting in a larger impact force.

### 3.3. Absorption Energy

**Figure 8:**
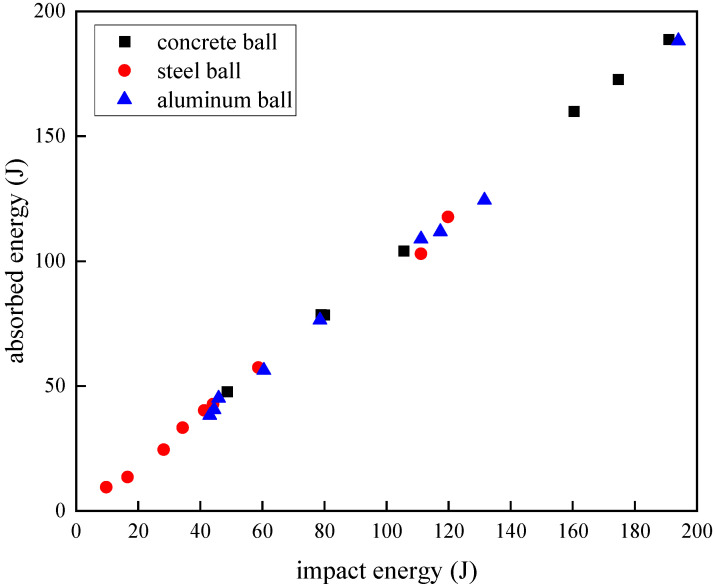
Absorbed energy under different impact energies.

Observing the projectile impact process, it was found that the displacement of the projectile in the vertical direction was very small relative to its movement in the horizontal direction during the time when the projectile hit the carbon fiber laminate and rebounded, so the change in the gravitational potential energy of the projectile in this process could be ignored. And the change in the thermal energy of the projectile during the impact was also very small. Then, according to the law of conservation of energy, without considering the gravitational potential energy of the projectile and the change in the thermal energy, the total absorbed energy of the laminate is the reduction in the kinetic energy of the projectile, and its expression is as follows:

(4) $$E_{t}=\frac{1}{2}m{v_{i}}^{2}-\frac{1}{2}m{v_{r}}^{2}$$

where *E_t_* is the total energy absorption of the laminate, *v_i_* is the projectile impact velocity, and *v_r_* is the remaining rebound velocity. The absorption energy calculation results of the laminate are recorded as shown in Table 5. The absorption energy of the carbon fiber composite laminate under different impact energies is plotted, and it could be seen that the larger the impact energy the greater the absorption energy under the condition that the laminate was not pierced, and its growth relationship was almost linear. In the case of the aluminum ball as a projectile, the absorption energy of the laminate calculated at the same impact energy was slightly smaller than that of the projectile of the other two materials. In fact, the aluminum ball would be slightly deformed after hitting the carbon fiber laminate, and the concrete ball and the steel ball were not deformed in the above impact velocity range, so the energy calculation formula of the aluminum ball is as follows:

(5) $$E_{ct}=\frac{1}{2}m{v_{i}}^{2}-\frac{1}{2}m{v_{r}}^{2}=E_{at}+U$$

where *E_ct_* is the calculated energy absorbed by the laminate, *E_at_* is the actual energy absorption, and *U* is the deformation energy of the aluminum ball. In the case of projectile deformation, theoretically, the total absorption energy calculated by the laminate was larger than the actual absorption energy, but the calculated value in Figure 8 was smaller than that of the non-deformed projectile impact case, which indicated that the actual energy absorption was even smaller, and for the impact objects with different deformation capabilities, the energy absorption characteristics of the carbon fiber laminate would show a slight difference.

## 4. Damage Response and Discussion

### 4.1. Failure Modes

The damage mode of carbon fiber composite laminates after high-velocity impact is complex, and understanding their damage characteristics is essential for the effective design and application of composite laminates. According to the experimental results, the damage response of the spherical projectile after impacting the carbon fiber specimen could be roughly divided into the following typical stages. The first was the elastic stage, where the projectile rebounded after hitting the carbon plate, and the laminate vibrated freely in the deflection direction, leaving no cracks, permanent indentations, and other visible damage on its surface and back. At this point, part of the kinetic energy lost by the projectile was dissipated through the elastic deformation generated by the laminate. Then, there was the first stage of laminate damage, where after the projectile rebounded, only small cracks or slight indentations were produced at the impact site, and the back side of the impact was flat without visible damage and protrusions; this stage was hereafter referred to as damage degree Ⅰ. In the second stage of laminate damage, referred to as damage degree Ⅱ, the surface of the laminate would produce obvious damage, such as cracks, crater indentation, interlayer delamination, fiber breakage, etc., and the back side of the impact began to produce protrusions, fiber pull-out, and other phenomena. In this case, the impact energy of the projectile would be dissipated in a number of ways. The spherical projectile impacted the laminate, and the incident pressure wave propagated along the thickness direction. The relatively minor damage at this stage was manifested as the compression failure of the laminate, and the local compacting at the impact site formed a circular crater, and the peripheral cracks of the crater extended along the cross shape. More severe damage was manifested by a fiber fracture on the impact side, as well as tear extraction and interlaminar delamination on the non-impact side, and the reflected tensile stress wave was an important cause of this injury. In the third stage of damage, referred to as damage degree Ⅲ, the laminate was broken down. The impact part of the projectile was compacted after it touched the laminate, and as the projectile gradually penetrated, the laminate generated the cross-shaped tear of the back fiber layer, and the broken fibers in the layer were continuously extruded from the gap to form a diamond-shaped protrusion. In the third stage, part of the initial kinetic energy was dissipated by the friction between the projectile and the laminate.

**Figure 9:**
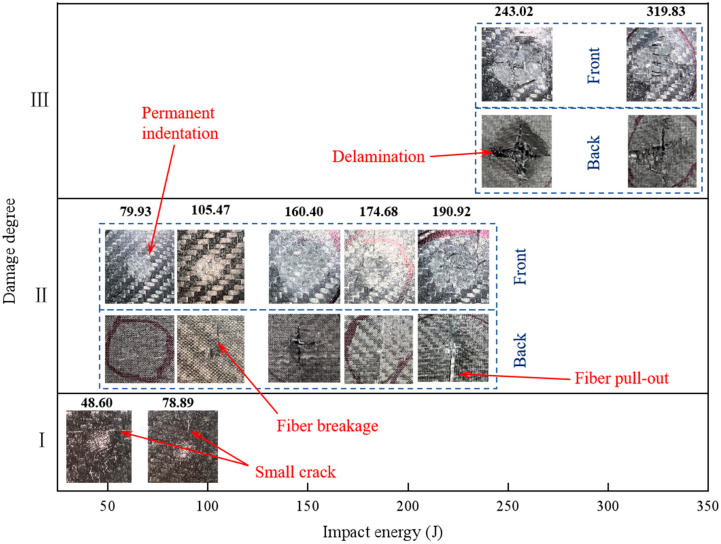
The 24 g concrete ball impact carbon fiber specimen’s impact side and non-impact side morphology.

Figure 9 illustrates the progressive damage evolution and failure patterns observed on the impact and distal surfaces of the laminate subjected to 24 g concrete projectile impact. At 48.60 J impact energy, incipient transverse cracking became apparent on the impacted surface. When the impact energy increased to 78.89 J, characteristic cruciform radial cracking patterns developed in the laminate structure. The specimen entered the secondary damage degree at 79.93 J, exhibiting permanent surface indentation accompanied by initial back side protrusion. Under 160.40 J impact loading, concentric crater damage features formed around the impact zone on the front surface, while a distinct conical deformation bulge emerged on the rear surface. At the threshold energy of 243.02 J, complete perforation occurred with the laminate transitioning to a tertiary failure stage, manifesting circumferential ring cracking on the impact surface and through-thickness conical fracture propagation in the laminate structure.

**Figure 10:**
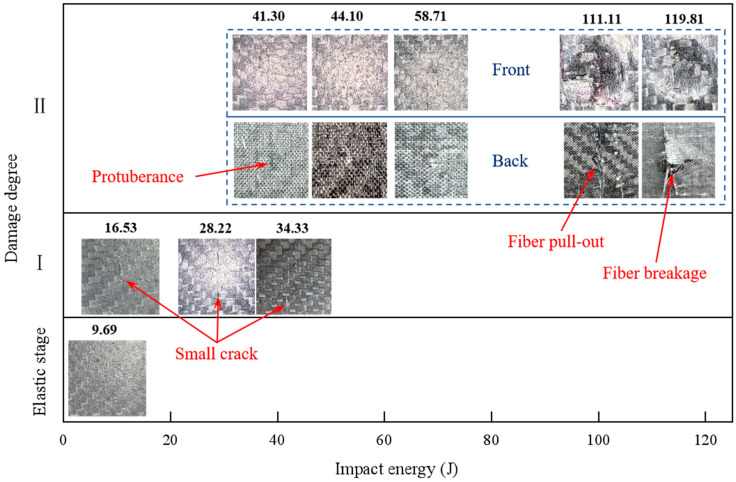
The 18 g steel ball impact carbon fiber specimen’s impact side and non-impact side morphology.

Figure 10 demonstrates the damage progression in the carbon fiber specimen under the 18 g steel projectile impact. At 9.69 J impact energy, the specimen remained in the elastic deformation stage without discernible surface damage. The subsequent energy increase to 16.53 J initiated primary damage characterized by fine transverse cracks. A transitional cruciform radial crack pattern emerged at 28.22 J, signaling advanced failure. When the impact energy was 41.30 J, the protrusion formed by fiber extrusion in the thickness direction began to appear on the back side of the laminate impact. When the energy reached 111.11 J, through-thickness fiber extrusion became evident, manifesting circumferential crack propagation that encircled the impact zone on the front surface, while fiber pull-out generated a through-thickness conical fracture on the distal side, accompanied by significant interlaminar delamination and ply separation.

**Figure 11:**
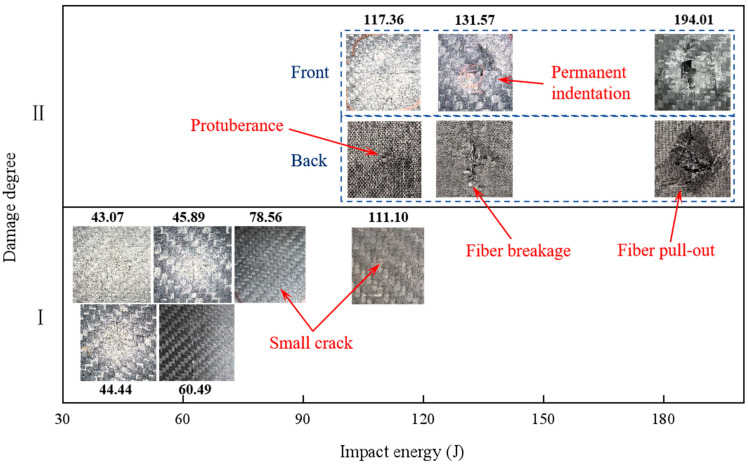
The 20 g aluminum ball impact carbon fiber specimen’s impact side and non-impact side morphology.

As illustrated in Figure 11, under impact testing with 20 g aluminum projectiles, the carbon fiber laminate exhibited progressive damage modes across increasing energy levels. At 43.07 J, the first stage of damage manifested as localized transverse cracking. Subsequent loading to 44.44 J initiated cross-shaped radial cracks within the composite structure. Energy escalation to 117.36 J induced fiber-bundle protrusion on the rear surface. When the impact energy reached 131.57 J, circumferential ring cracks formed on the impacted surface accompanied by subsurface cone crack propagation through multiple plies. The critical energy threshold of 194.01 J resulted in severe rear-surface bulging without full perforation.

Based on the above experimental results, the impact energy required for carbon fiber laminates to enter each damage degree was not the same under projectiles with different materials. When the carbon fiber plate entered the second degree of damage, the impact energy required for aluminum balls, concrete balls, and steel balls was 117.36 J, 79.93 J, and 41.30 J, respectively. The disparity in the threshold for the beginning of the second damage degree was predominantly attributed to variations in projectile geometry and material-specific deformation. The steel projectiles exhibited a slightly smaller diameter compared to that of the concrete and aluminum projectiles. This dimensional discrepancy resulted in a diminished contact area during impact, thereby generating intensified localized stress concentrations for the steel projectiles. The elevated stress levels likely lowered the energy threshold required to initiate damage degree II in the laminate. Conversely, aluminum projectiles exhibited plastic deformation that induced energy dissipation. This inherent energy absorption characteristic consequently increased the energy threshold required for damage degree II progression when compared to that of the concrete projectiles.

However, for spherical projectiles, the damage development of carbon fiber laminates showed regularity, starting from the appearance of small cracks on the surface, and the location of the cracks mostly occurred in the cross azimuth centered on the impact point (in most cases, the crack extension range was much larger than the diameter of the impact contact surface and the projectile’s diameter). Then, the indentation or impact crater appeared on the surface of the laminate; the fiber protrusions, pull-out, and tears appeared on the back side of the impact; the layers were delaminated; the fiber blocks were broken; and finally the laminate was pierced.

### 4.2. Crack Range

**Figure 12:**
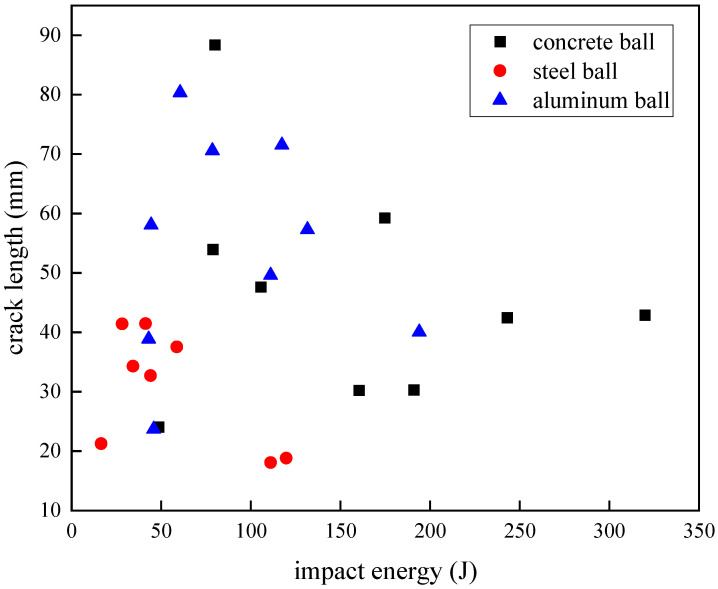
Crack length under different impact energies.

The crack extension length is a manifestation of the degree of failure of the composite laminate, and the experimental results of all the cracks on the impact side were observed. The crack length was defined as the maximum diameter of the crack measured in the standard ASTM D7136/D7136M-20 [38], the specific data are shown in Table 7, and Figure 12 shows the crack-length response of the carbon fiber specimen when the projectile of three materials was used as the impact object. The crack length had a phased change trend with the increase in impact energy. Under the condition of the steel ball impact, the crack length on the surface of the carbon fiber specimen first increased with the increase in impact energy and reached the maximum length of 41.48 mm when the impact energy was 41.3 J, and then the crack length decreased. When the impact energy of the aluminum ball was 60.49 J, the maximum crack length of the laminate was 80.35 mm. Under the impact condition of concrete balls, when the impact energy increased to 79.93 J, the crack length reached the maximum value of 88.4 mm, and it then decreased. However, when the impact energy reached 243.02 J, the crack length increased slightly, and it could be seen that there were two near-horizontal samples in Figure 12, with crack lengths of 42.45 mm and 42.9 mm, respectively.

Observing the crack-length performance of the carbon fiber laminate on the projectiles of the three materials, it was found that the change trend of the impact energy increased first and then decreased in the process of gradual increase, among which the crack-length range of the steel ball with the smaller volume was obviously smaller under the impact, while the experimental results of the concrete ball and the aluminum ball had a considerable overlap range below the impact energy of 200 J, and the volume of the two projectiles was actually very similar. Within the experimental findings in our study, the post-impact crack propagation dimensions in the laminates are predominantly governed by the spherical projectile’s diameter. Specifically, projectiles with larger diameters create expanded contact zones during penetration, thereby generating increases in crack length.

Comparing and observing the failure mode of the carbon fiber laminates, the impact energy increased but the cracks did not continue to increase because the laminate began to appear with more damage other than the cracks, such as surface fiber fractures and fragmentation, or back fiber protrusion damage, and the laminate absorbed the impact kinetic energy of the projectile in other forms, so the crack range would not expand indefinitely. When the concrete ball was impacted with energy above 243.02 J, the carbon fiber laminate specimens were all pierced, indicating that the crack range of the laminate after the impact tended to be stable under the perforation state.

**Figure 13:**
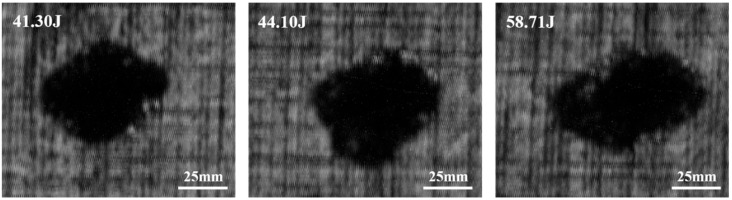
C-scan delamination image of carbon fiber laminates.

To elucidate the mechanism underlying the observed reduction in crack length with increasing impact energy beyond a critical threshold, three experimental groups exhibiting similar macroscopic damage characteristics (specifically, secondary-damage-degree manifestations with visible back side protuberances) were subjected to water-immersed C-scan analysis, the scan results are shown in Figure 13. The investigation focused on carbon fiber laminates impacted by 18 g steel projectiles at energy levels of 41.30 J, 44.10 J, and 58.71 J. C-scan quantification revealed delamination areas of 2450 mm^2^, 2737.5 mm^2^, and 2850 mm^2^, respectively, corresponding to measured crack lengths of 41.48 mm, 32.70 mm, and 37.55 mm. This inconsistent relationship between impact energy and crack propagation demonstrates that enhanced energy absorption through progressive delamination expansion (evidenced by increasing delamination area with higher impact energies) effectively dissipates impact energy, thereby suppressing further crack extensions despite elevated energy inputs.

### 4.3. Penetration

**Figure 14:**
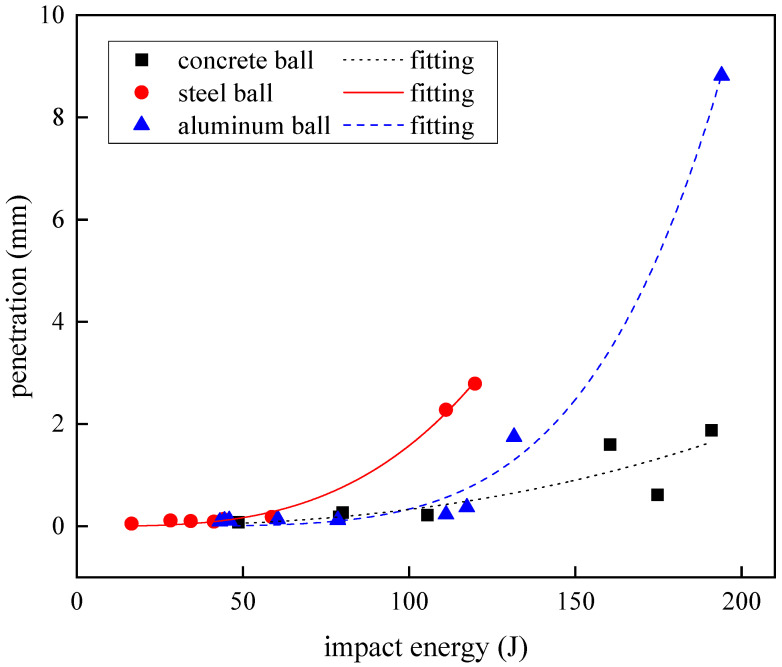
Penetration under different impact energies.

In practical engineering applications, when the carbon fiber laminate is used as a protective structure, the displacement penetration in the thickness direction after impact is an intuitive index to evaluate the impact resistance of the laminate. The penetration depth obtained by the experiment was recorded in Table 7. Figure 14 shows the relationship between the impact energy of the three spherical projectiles and the amount of penetration of the carbon fiber laminate displacement. At the lower impact energy (below 100 J), the penetration amount of the laminate was very small, and the increase with the increase in impact energy was also small, and the samples of projectiles of different materials fell almost at the same horizontal trajectory, which meant that the projectile material had no effect on the penetration resistance of the carbon fiber laminate in the thickness direction at this stage. When the impact energy continued to increase to more than 100 J, the penetration depth began to increase greatly. In the experimental data, the displacement penetration exceeded the thickness of the laminate, which was due to the fracture of the carbon fiber during the impact process, and the fiber bundle was squeezed backward by the projectile, which quickly formed a protrusion blockage on the back side of the impact, but the projectile failed to pierce the laminate and rebounded and left the penetration results beyond the thickness of the laminate. By observing the change in penetration, it was considered that there was an accelerating nonlinear growth trend. In order to study this more intuitively, the Allometic1 function was used to fit it, and the expression of the function was $y=ax^{b}$, the fitting parameters of the three curves are recorded in Table 8. As shown in Figure 14, the projectiles of different materials showed different penetration growth when they had large impact energy. The growth rate of the penetration of steel projectiles changed earlier, and the penetration displacement of steel projectiles was the largest compared with that of concrete and aluminum balls under the same impact energy. After the impact energy of the aluminum projectile was 131.57 J, the penetration amount increased rapidly. When the penetration of the concrete projectile began to increase dramatically, the impact energy was the largest among the three materials, and the growth rate of its penetration was small and slow, which was also reflected by the small b value of its fitting curve.

The fitting curves of the relationship between the penetration depth and impact energy of three kinds of projectors were observed, and steel projectiles exhibiting higher rigidity and reduced diameters demonstrate superior penetration depth in laminated composites compared to larger-diameter counterparts under equivalent impact energy conditions. This phenomenon is attributed to the localized stress concentration resulting from a diminished contact surface area. However, experimental observations reveal a counterintuitive behavioral pattern when comparing plastically deformable aluminum projectiles with concrete projectiles of comparable diameters. Contrary to conventional expectations, the aluminum projectiles exhibiting plastic deformation achieved greater penetration depths than their non-deforming concrete counterparts under identical impact energy conditions. Reference [22] demonstrates that the post-impact failure analysis of FRP laminates reveals different through-thickness damage mechanisms: the frontal region exhibits extrusion failure, the middle zone shows matrix shear failure, while the rearward section manifests combined tensile fractures and interlaminar delamination phenomena. During aluminum projectile impact, the plastically deformed contact interface maintains its geometric acuity, generating intensified shear stress concentrations within the carbon-fiber-reinforced polymer (CFRP) laminate. This localized stress state promotes the development of frontal compressive crushing and middle shear failure mechanisms. Thus, the penetration depth of the aluminum projectiles is greater than that of the concrete projectiles.

### 4.4. Response Relevance

**Figure 15:**
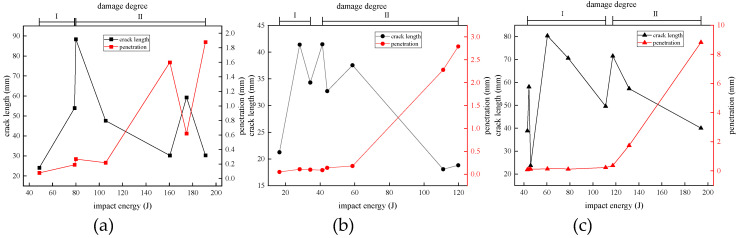
Damage response of (**a**) concrete, (**b**) steel, and (**c**) aluminum projectile.

The correlation between the impact response of carbon fiber laminates under the impact of spherical projectiles was analyzed. It can be seen from Figure 15 that when the carbon fiber specimen was in the first damage stage, its damage was mainly reflected in the growth of the crack length on the impact front, and the penetration amount in this stage was very small and had almost no change trend. When the specimen entered the second stage of damage, the visible damage was marked on the back side of the impact, the crack length on the surface of the impact side of the carbon fiber plate gradually decreased, the penetration depth increased significantly, and the damage performance changed from the crack extension on the surface to the penetration in the thickness direction, indicating that when the carbon fiber plate was subjected to high-velocity impact, the later stage of the damage was mainly due to the tensile deformation, fractures, fragmentation, and interlayer delamination of the fiber to absorb energy instead of crack propagation.

## 5. Conclusions

This investigation systematically examined the high-velocity-impact behavior of carbon fiber composite laminates through gas gun experiments, with an emphasis on impact force quantification and damage progression.

1. The velocity and acceleration of the projectile were derived from high-speed photographic image sequences, enabling the calculation of the average impact force exerted on the carbon fiber laminate under high-speed-impact conditions. Analysis of the force data revealed that the average impact force exhibited a positive correlation with projectile impact energy, albeit with a diminishing rate of increase. Notably, deformable projectiles demonstrated lower average impact forces compared to those of their rigid counterparts during impact events, a phenomenon attributed to the prolonged contact duration resulting from their plastic deformation.
2. The damage development of the carbon fiber laminate after being impacted by a spherical projectile started from the generation and extension of cracks on the impact surface, forming permanent indentations on the front surface; the back side produced fiber protrusions and fractures; the layers were delaminated; the fibers were broken in a lumpy manner; and finally the laminate was pierced. For different projectile materials, the impact energy required for carbon fiber laminates to produce the same degree of damage was different.
3. With the increase in impact energy, the crack length of the carbon fiber laminate increased first and then decreased, and it then flattened after penetration, and the size of the overall crack range had a certain relationship with the volume of the spherical projectile. In the case of non-perforation, the penetration depth increased allometrically with the increase in impact energy, its growth rate became larger and larger, and its change trend correlated with the crack range, and when the penetration amount began to increase rapidly, the crack range was in the decreasing stage.

## Figures and Tables

**Table 1:** Carbon fiber layup information.

Number	Carbon Fiber Type	K-Number	Layup Angle
1	T300	3 K	45°
2	T700	12 K	0°
3	T700	12 K	45°
4	T700	12 K	0°
5	T700	12 K	45°
6	T700	12 K	0°
7	T300	3 K	45°
8	T700	12 K	0°
9	T700	12 K	45°
10	T700	12 K	0°
11	T700	12 K	45°
12	T700	12 K	0°
13	T300	3 K	45°

**Table 2:** Impact projectile parameters.

Material	Quality (g)	Diameter (mm)	Density (g/cm^3^)	Strength (MPa)	
concrete	24	25	2.93	50	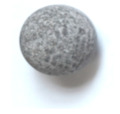
stainless steel	18	16.5	7.65	485	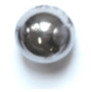
aluminum	20	24	2.76	286	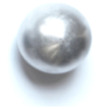

Note. The strength of concrete in the column of strength refers to compressive strength, the strength of stainless refers to tensile strength, and the strength of aluminum refers to yield strength.

**Table 3:** Experimental results.

Projectile	Number of Groups	Impact Velocity Range	Diagram of the Impact Sequence
Concrete balls	9	229.09–587.72 km/h	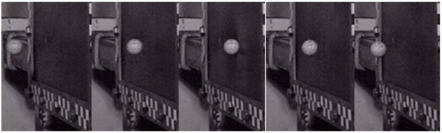
Steel balls	9	118.13–415.37 km/h	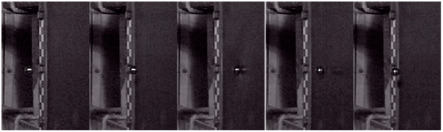
Aluminum balls	9	236.25–501.43 km/h	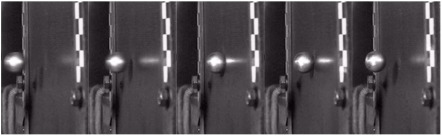

**Table 4:** Projectile velocity distribution.

	Velocity km/h	115–195	195–235	235–275	275–315	315–355	355–395	395–435	435–475	475–515	515–595
Projectile											
Concrete			√		√	√		√	√	√	√
Steel		√	√	√	√			√			
Aluminum				√	√	√	√	√		√	

**Table 5:** Impact force and energy absorption data.

Projectile	Initial Velocity (km/h)	Residual Velocity (km/h)	Average Impact Force (kN)	Energy Absorption (J)
Concrete balls	229.09	30.86	5.81	47.71
	291.89	17.53	6.79	78.61
	293.81	39.09	6.80	78.52
	337.50	38.16	7.23	104.12
	416.21	22.02	7.48	159.95
	434.34	46.17	7.38	172.70
	454.08	48.69	7.75	188.72
Steel balls	118.13	17.03	3.37	9.49
	154.29	65.74	4.47	13.53
	201.60	73.04	5.58	24.52
	222.35	37.80	5.18	33.34
	243.87	38.67	5.67	40.26
	252.00	44.08	5.85	42.75
	290.77	43.70	6.65	57.39
	400.00	108.07	7.36	103.00
	415.37	55.57	7.33	117.67
Aluminum balls	236.25	78.75	5.81	38.28
	240.00	71.32	4.96	40.52
	243.87	32.10	5.08	45.09
	280.00	74.12	5.57	56.25
	319.09	53.38	6.89	76.36
	379.46	53.79	6.95	108.87
	390.00	84.73	6.06	111.82
	412.94	96.40	6.41	124.40
	501.43	86.41	6.95	188.24

**Table 6:** ExpAssoc function fitting parameters for impact force.

Projectile	Equation	*y*_0_	*A*_1_	*t*_1_	*A*_2_	*t*_2_	*R* ^2^
concrete	*y* = *y*_0_ + *A*_1_ × (1 − *exp*(−*x*/*t*_1_)) + *A*_2_ × (1 − *exp*(−*x*/*t*_2_))	−2.77184	9.66332	23.29609	9.56654	23.40645	0.97868
steel		−14.23242	17.55073	3.05128	4.4336	49.88058	0.97327
aluminum		−13.49309	13.42403	0.19683	6.80662	28.75694	0.67767

**Table 7:** Damage behavior data.

Projectile	Impact Energy (J)	Crack Length (mm)	Penetration Depth (mm)
concrete	48.60	24.07	0.08
	78.89	53.95	0.19
	79.93	88.4	0.27
	105.47	47.62	0.22
	160.40	30.26	1.60
	174.68	59.27	0.62
	190.92	30.33	1.88
	243.02	42.45	perforation
	319.83	42.9	perforation
steel	16.53	21.26	0.05
	28.22	41.41	0.11
	34.33	34.31	0.10
	41.30	41.48	0.09
	44.10	32.70	0.14
	58.71	37.55	0.18
	111.11	18.08	2.28
	119.81	18.82	2.79
aluminum	43.07	38.86	0.09
	44.44	58.07	0.11
	45.89	23.69	0.12
	60.49	80.35	0.14
	78.56	70.59	0.12
	111.10	49.60	0.23
	117.36	71.52	0.37
	131.57	57.28	1.75
	194.01	40.04	8.82

**Table 8:** Allometic1 function fitting parameters for penetration.

Projectile	*a*	*b*	*R* ^2^
concrete	5.4517 × 10^−10^	0.6087	0.9516
steel	5.3023 × 10^−7^	3.2367	0.9969
aluminum	3.1305 × 10^−9^	4.1349	0.9229

## Data Availability

The original contributions presented in this study are included in the article. Further inquiries can be directed to the corresponding author.
